# Bioactive Lipids of Marine Microalga *Chlorococcum* sp. SABC 012504 with Anti-Inflammatory and Anti-Thrombotic Activities

**DOI:** 10.3390/md19010028

**Published:** 2021-01-10

**Authors:** Katie Shiels, Alexandros Tsoupras, Ronan Lordan, Constantina Nasopoulou, Ioannis Zabetakis, Patrick Murray, Sushanta Kumar Saha

**Affiliations:** 1 Shannon Applied Biotechnology Centre, Limerick Institute of Technology, Moylish Park, V94 E8YF Limerick, Ireland; Katie.Shiels@lit.ie (K.S.); Patrick.Murray@lit.ie (P.M.); 2 Department of Biological Sciences, University of Limerick, V94 T9PX Limerick, Ireland; Alexandros.Tsoupras@ul.ie (A.T.); Ioannis.Zabetakis@ul.ie (I.Z.); 3 Health Research Institute, University of Limerick, V94 T9PX Limerick, Ireland; 4 Bernal Institute, University of Limerick, V94 T9PX Limerick, Ireland; 5 Institute for Translational Medicine and Therapeutics, Perelman School of Medicine, University of Pennsylvania, Philadelphia, PA 19104, USA; ronan.lordan@pennmedicine.upenn.edu; 6 Department of Food Science and Nutrition, School of the Environment, University of the Aegean, GR 81400 Myrina, Lemnos, Greece

**Keywords:** marine microalga, *Chlorococcum* sp., bioactive lipid, anti-inflammatory, antithrombotic, platelets

## Abstract

Microalgae are at the start of the food chain, and many are known producers of a significant amount of lipids with essential fatty acids. However, the bioactivity of microalgal lipids for anti-inflammatory and antithrombotic activities have rarely been investigated. Therefore, for a sustainable source of the above bioactive lipids, the present study was undertaken. The total lipids of microalga *Chlorococcum* sp., isolated from the Irish coast, were fractionated into neutral-, glyco-, and phospho-lipids, and were tested in vitro for their anti-inflammatory and antithrombotic activities. All tested lipid fractions showed strong anti-platelet-activating factor (PAF) and antithrombin activities in human platelets (half maximal inhibitory concentration (IC_50_) values ranging ~25–200 μg of lipid) with the highest activities in glyco- and phospho-lipid fractions. The structural analysis of the bioactive lipid fraction-2 revealed the presence of specific sulfoquinovosyl diacylglycerols (SQDG) bioactive molecules and the HexCer-t36:2 (t18:1/18:1 and 18:2/18:0) cerebrosides with a phytosphingosine (4-hydrosphinganine) base, while fraction-3 contained bioactive phosphatidylcholine (PC) and phosphatidylethanolamine (PE) molecules. These novel bioactive lipids of *Chlorococcum* sp. with putative health benefits may indicate that marine microalgae can be a sustainable alternative source for bioactive lipids production for food supplements and nutraceutical applications. However, further studies are required towards the commercial technology pathways development and biosafety analysis for the use of the microalga.

## 1. Introduction

Marine microalgae are the pioneering photosynthetic organisms with significant morphological, genetic, and biochemical diversity. They are playing an important role in the biosphere, supplying nutrition to both aquatic and terrestrial food chains. Microalgae are ubiquitously distributed throughout the biosphere and exposed to high-oxygen and free-radical stresses, which has led to the evolution of efficient anti-oxidative defence mechanisms [1]. Marine algae living in the competitive harsh environments have developed specialised defence strategies by biosynthesising chemically and structurally diverse compounds through different metabolic pathways [2]. Microalgae are the “treasure house”, as rich sources of proteins, essential fatty acids, carbohydrates, pigments, vitamins, minerals, and several other bioactive molecules, with anti-oxidant, anti-inflammatory, antithrombotic, anti-bacterial, anti-diabetic, anti-fungal, anti-viral, anti-parasitic, anti-proliferative, anti-elastase, anti-trypsin, anti-chymotrypsin, angiotensin I-converting enzyme inhibitory (ACE-inhibitory), myofibroblast differentiation inducing, hepatic fibrosis inhibitory, etc. [3,4,5,6,7].

Inflammation and thrombosis are implicated in several chronic disorders, such as cardiovascular diseases, cancer, and persistent infections, such as the new coronavirus disease 2019 (COVID-19) pandemic [8,9,10,11]. Platelet-activating factor (PAF) and thrombin are key mediators of the inflammatory and thrombo-inflammatory manifestations implicated in the onset and/or development of such disorders [9]. These mediators activate several pathways and cells of the immune system, including platelets, through specific receptors in the membranes of these cells. The abnormal activation of human platelets is implicated in thrombosis and/or pathological bleeding although these cells are critically involved in normal haemostasis [9,12,13]. Recent research has emphasised on the beneficial effects of marine polar lipids (PL) towards inflammation related disorders, through an array of beneficial bioactivities. These beneficial effects were reported due to their anti-inflammatory and antithrombotic activities through mechanisms, such as the inhibition of the PAF and thrombin related pathways, and the modulation of PAF metabolism towards reducing PAF-levels [8,9,14,15].

Nowadays, microalgae are considered one of the most promising feedstocks for sustainable source of various commodities for food, feed, cosmetics, and other applications [5,6]. Several microalgae have already been commercially produced for their use as nutrient-rich food, feed, and health promoting bioactive compounds [7]. However, many more microalgae are yet to be commercialised with their already known bioactivities, and on-going research activities may further identify microalgae as new potential sources for bioactive compounds. *Chlorococcum* is one such microalga that has not been thoroughly researched for commercial exploitation, except there are few academic studies related to carotenoid and lipids analysis. *Chlorococcum* is unicellular green microalgae of Chlorophyceae, found both in freshwater and marine habitats. The cells of microalga are spherical or slightly oblong with varied cell sizes, which may live as solitary or in irregular clump of cells. This green microalga has a single cup-shaped, parietal chloroplast with a single pyrenoid [7]. *Chlorococcum* was known to produce carotenoids: astaxanthin, adonixanthin, canthaxanthin, β-carotene, lutein, and ketocarotenoids [16,17]. Generally, during carotenogenesis process microalgae accumulates lipids with specific fatty acids production that esterifies with carotenoids and, thus, helps the organism survive the stress conditions [18]. In a study for biodiesel production, it was found that a freshwater *Chlorococcum* sp. RAP13 was well adapted to marine growth condition and can accumulate up to 38% lipids (of dry weight biomass) under heterotrophic condition [19].

The marine *Chlorococcum* sp. lipids have not been tested yet towards the inflammatory and thrombotic pathways of PAF and thrombin. Therefore, in this manuscript, we have characterised the lipid fractions of marine microalga *Chlorococcum* sp. for anti-PAF and antithrombin activities with a view that the microalga can be an alternative sustainable source for the above bioactivities. 

## 2. Results and Discussion

### 2.1. Bioactivity of Lipid Fractions

Within the present study, the antithrombotic potency of bioactive lipid fractions of *Chlorococcum* sp. was evaluated by assessing their putative inhibitory effect on aggregation of human platelets induced by the thrombotic and inflammatory mediators, PAF and thrombin, while the overall structures and fatty acid composition of the most bioactive lipids were elucidated through LC-electrospray ionization (ESI)-MS analysis. Such an experimental approach of fractionating bioactive lipids in classes, evaluating bioactivity against PAF and thrombin in platelets and identifying bioactive molecular species in each lipid class by LC-ESI-MS, has previously been effectively used in other microalgae, cyanobacteria [3,20], and other marine sources [21,22]. Table 1 shows the comparative lipid content of various microalgae, which indicates that the source of microalgae as well as their growth conditions may reflect on their lipid content. The microalga *Chlorococcum* sp. SABC 012504 of this study possess relatively medium range of lipid content as per the comparative table. However, it may be possible to further enhance the lipid content of this microalga as that of other closely related microalgae tested (Table 1).

**Figure 1:**
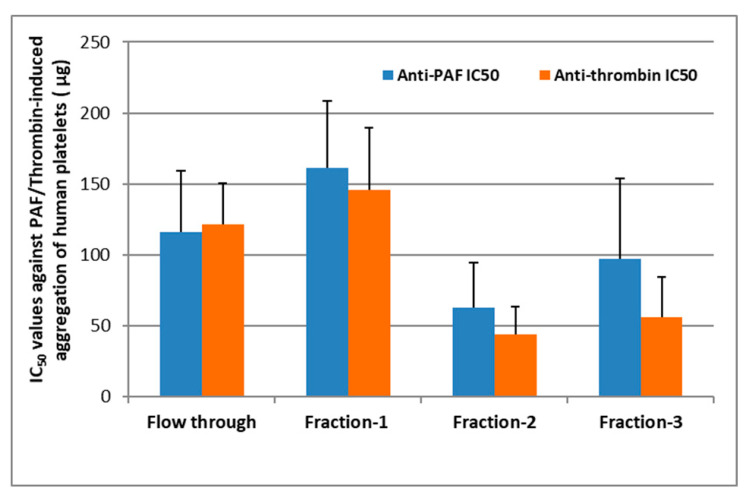
Microalgal bioactive lipid fractions showing the inhibitory effect towards the platelet-activating factor (PAF) and thrombin pathways of platelet aggregation in human platelet-rich plasma (hPRP). IC_50_ values reflect the inhibitory strength of each lipid fractions towards PAF/thrombin-induced platelet aggregation in hPRP and is expressed as mean values of μg of lipids in the aggregometer cuvette that causes 50% of inhibition on PAF/thrombin-induced platelets aggregation in hPRP ± SD.

More specifically, lipid extracts from *Chlorococcum* sp. were fractionated as per the method of Saha et al. [18] and found to share similar features with the previously reported outcomes for this fractionation; fraction-1 contained the more neutral lipids, fraction-2 the glycolipids, and fraction-3 the phospholipids of this lipid extract. Each lipid fraction obtained was further assessed for its ability to inhibit human platelet aggregation induced by PAF and thrombin as previously described [22]. All fractions assessed exhibited strong anti-PAF and antithrombin activities in human platelets, with half maximal inhibitory concentration (IC_50_) values within the range of ~25–200 μg of lipid bioactives present in the hPRP suspension (Figure 1).

It should also be stressed out that the lower the IC_50_ value for a lipid sample/bioactive the stronger its inhibitory effect against human platelet aggregation induced either by PAF or by Thrombin. Thus, fractions 2 and 3 corresponding to the glycolipids and phospholipids of *Chlorococcum* sp. exhibited the strongest anti-PAF and antithrombin activities, while neutral lipids of fraction-1 exhibited the weakest bioactivities against PAF and thrombin. The flow through sample exhibited an intermediate activity against both PAF and thrombin, suggesting that some lipids from the initial lipid extract may be soluble in the flow through and have co-migrated to this fraction during the initial steps of the fractionation procedure.

The IC_50_ values of fractions 2 and 3 against both PAF and thrombin were found to be within the range of approximately 30–100 μg against the PAF pathway and 25–80 μg against the thrombin pathway of human platelet aggregation, respectively, which are within the same order of magnitude with those of bioactive phospholipids’ and glycolipids’ fractions derived from other marine sources, such as salmon, herring, and boarfish [21,22,30]. Moreover, both antithrombin effects of fractions 2 and 3 were found to be statistically significant stronger than the antithrombin effect of the neutral lipids of fraction-1, while only the anti-PAF effect of fraction-2 was statistically significant stronger than that of the neutral lipids of fraction-1 (*p* < 0.05 in all these comparisons). However, no statistically significant difference was observed between the IC_50_ values of fractions 2 and 3 against both PAF and thrombin (*p* > 0.05 in all these comparisons). These results further suggest that the more polar lipids of *Chlorococcum* sp. are more bioactive against the PAF and the thrombin pathways of inflammation and thrombosis, rather than their neutral lipid compounds, which comes also in accordance with previously reported outcomes for polar lipids from other microalga and cyanobacteria [3,4,20], but also with bioactive polar lipids derived from other natural sources with anti-inflammatory and antithrombotic properties [9].

Nevertheless, the observed strong antithrombotic properties of the bioactive glycolipids and phospholipids in lipid fractions 2 and 3 of *Chlorococcum* sp. seem to be attributed to a synergism of highly bioactive polar lipid molecules that coexist in *Chlorococcum* sp. For this reason, apart from the fatty acid composition, the overall structures of bioactive molecules present in these most bioactive lipid fractions were also elucidated through LC-MS analysis as previously described [21,22,31].

### 2.2. Fatty Acids Analysis

A total of 15 fatty acids were identified among the various lipids fractions, of which eight were saturated fatty acids (SFAs) and seven were unsaturated fatty acids (UFAs) (Table 2). Within each fraction, a maximum of six SFAs and seven UFAs were, respectively, detected in saponified samples of fraction-3 (phospholipids) and fraction-2 (glycolipids). A minimum of two SFAs were detected in unsaponified sample of fraction-3 (phospholipids) and no UFAs were detected in three unsaponified samples of unbound flow through (FT), fraction-2 (glycolipids), and fraction-3 (phospholipids). In general, all saponified samples showed more numbers and/or amounts of detectable fatty acids compared to the corresponding unsaponified lipid samples as expected. This indicates newly appeared fatty acids and/or their increased peak abundance are due to their complex nature in unsaponified form of lipids. Additionally, the number of UFAs were increased in all saponified lipid samples indicating their involvement in complex formation possibly for their biological function/activity. Of the UFAs, the relative content of oleic acid (C18:1 (OA)) was highest, followed by linoleic acid (C18:2 (LA)) and linolenic acid (C18:3 (ALA (α-LA) /GLA (γ-LA)) in all saponified samples. While, UFA gadoleic acid (C20:1) was found only saponified samples of FT (unbound lipid), fraction-1 (neutral lipid), and fraction-2 (glycolipid).

### 2.3. Bioactive Lipids

**Figure 2:**
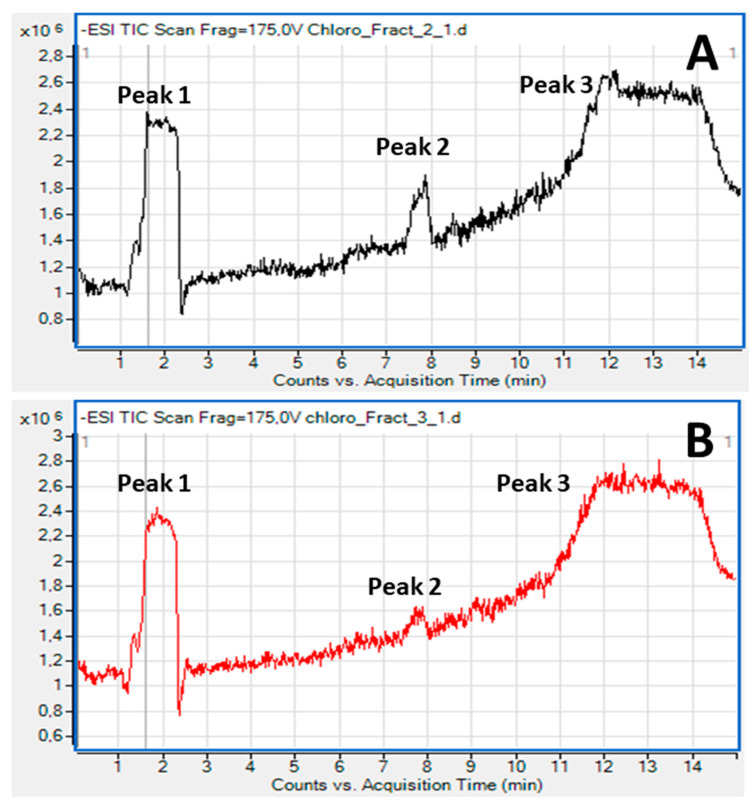
Representative chromatograms of the LC-MS analysis of the most bioactive lipid fractions 2 (**A**) and 3 (**B**) for glycolipids and phospholipids of *Chlorococcum* sp., respectively.

The overall structures of the most bioactive lipid species in fractions 2 and 3 of lipid extracts of *Chlorococcum* sp. were elucidated by LC-MS. During the LC-MS analysis, the separation of the lipid molecules in each fraction by HPLC was based on the length of the nonpolar acyl- or alkyl-groups in combination with their degree of unsaturation by using a C18 reverse-phase column. Characteristic chromatograms and relative peaks of this analysis are shown in Figure 2.

Moreover, by applying Q-TOF mass spectrometry, simultaneously with the HPLC separation of the lipid molecules in specific peaks, unique MS data were obtained leading to complete structural elucidations for each lipid molecule abundant in these peaks in both bioactive lipid fractions 2 and 3. The characterization of these molecules was based on the acquired *m*/*z* values of the demethylated negative ions (M–CH_3_)^−^ for phosphatidylcholine (PC) and sphingomyelin (SM) molecules and the dehydrogenated negative ions (M–H)^−^ for phosphatidylethanolamine (PE) molecules and for all the other glycosphingolipids (e.g., ceramides and cerebrosides) and other sulfoglycolipids found in these fractions, while further verification was obtained by using LIPID MAPS: Nature Lipidomics Gateway (www.lipidmaps.org) based on the lowest delta values during identification in combination with their fatty acids contents that were acquired by the LC-MS analyses of the free fatty acids (FFA) derived by the saponification of these lipid fractions.

**Figure 3:**
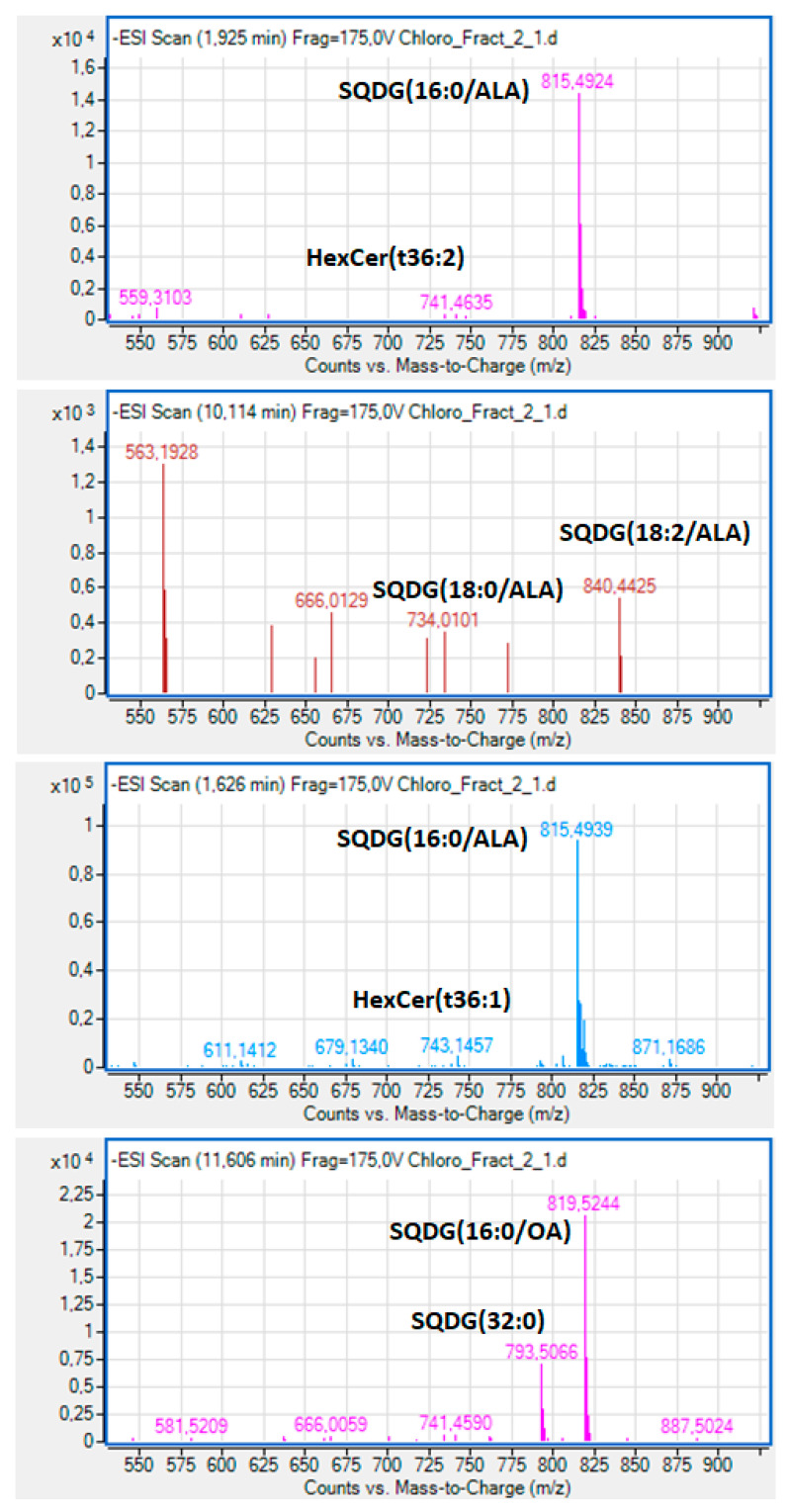
Representative mass spectra and proposed structures of the bioactive polar lipid molecules identified in the bioactive polar lipid fraction 2 of *Chlorococcum* sp., with strong anti-inflammatory and antithrombotic properties against the inflammatory and thrombotic mediators PAF and thrombin in human platelets. SQDG = sulfoquinovosyl diacylglycerols; HexCer(t36:2) and HexCer(t36:1) = glycosphingolipids (cerebrosides) bearing one hexose moiety (glucose or galactose) at their polar head, with a (t) phytosphingosine (4-hydrosphinganine) base; ALA = α-linolenic acid (18:3 n-3); OA = oleic acid (18:1).

More specifically, during the analysis of lipid molecules in fraction-2, it was found that the dominant bioactive polar lipid molecule eluted within peak-1 of Figure 2A was a sulfonic-acid containing polar glycolipid belonging to the family of sulfoquinovosyl diacylglycerols (SQDG) (Figure 3), bearing the omega 3 polyunsaturated fatty acid (n-3 PUFA) α-Linolenic acid (ALA: 18:3, n-3) at the *sn*-2 position of its glycerol backbone; SQDG (16:0/ALA). Other SQDG molecules bearing ALA at the *sn*-2 position of their glycerol backbones were also found to be eluted just before peak 3 of Figure 2A, like the SQDG (18:2/ALA) (Figure 3), while a SQDG bearing the monounsaturated fatty acid (MUFA) oleic acid (OA: 18:1) at its *sn*-2 glycerol backbone, SQDG (16:0/18:1) (Figure 3), was eluted in peak 3 of Figure 2A, along with a more saturated SQDG (16:0/16:0) molecule being co-eluted in the same peak with similar retention time (Figure 3).

SQDG are natural sulfoglycolipids found in all photosynthetic plants, cyanobacteria, and algae, and have been shown to have anti-inflammatory activities against the PAF-pathway through an antagonistic effect on PAF-receptor in rabbit platelets and in human neutrophils, but also through reducing PAF-levels by inhibiting PAF-biosynthesis [4,32]. Based on such outcomes, a European patent has been approved for the use of this glycolipid as a new PAF-receptor antagonist for the prophylaxis or treatment of inflammatory skin diseases, especially psoriasis [33]. Thus, the presence in abundance of such SQDG polar lipid molecules in the fraction-2, and especially those SQDG bearing the bioactive fatty acids n-3 ALA and the OA at the *sn*-2 position of their structure, provide an explanation of the strong anti-PAF and antithrombin activities of the polar lipid fraction-2 of *Chlorococcum* sp. that were observed in the present study in human platelets.

Furthermore, lower amounts of specific bioactive cerebrosides with a phytosphingosine (4-hydrosphinganine) base (t) and 1 hexose moiety (glucose/galactose), such as the HexCer(t36:1) and HexCer(t36:2), were also present in peak 1 (Figure 2A) of this bioactive polar lipid fraction-2 of *Chlorococcum* sp. (Figure 3). The presence of such bioactive glycosphingolipids (cerebrosides and ceramides) bearing one hexose moiety (glucose or galactose) at their polar head, with various bases at the sphingo-backbone (sphingosine, sphinganine, deoxysphinganine, 4,8-sphingodienine and phytosphingosine) in this lipid fraction with strong anti-PAF and antithrombin effects against platelet aggregation, comes in accordance with previously identified similar structures in other cyanobacteria lipid extracts, with both antagonistic and agonistic inhibitory effects against the PAF pathway [3,20], and further explains the bioactivities observed in fraction 2.

Moreover, the presence of such bioactive SQDG and glycosphingolipid molecules in the bioactive polar lipid fraction 2 of *Chlorococcum* sp. with strong anti-inflammatory and antithrombotic properties, may be of great importance for reducing platelet activation and thus the risk for cardiovascular diseases (CVD) and other inflammation-related chronic disorders, including cancer, since such molecules have been reported to possess strong antitumor properties [34,35]. In the present study, in the fraction 2 of *Chlorococcum* sp. we identified molecules with similar/identical structures, such as specific SQDG bioactive molecules and the HexCer-t36:2 (t18:1/18:1 and 18:2/18:0) cerebrosides with a phytosphingosine (4-hydrosphinganine) base, which are presented in Figure 3. Since PAF and thrombin are also implicated in cancer and tumour-related metastatic manifestations [8,9] it is possible that the antitumour effects of such SQDG and cerebroside molecules may also be related to their observed strong anti-PAF and antithrombin effects. However, further studies are needed to support such a notion.

**Figure 4:**
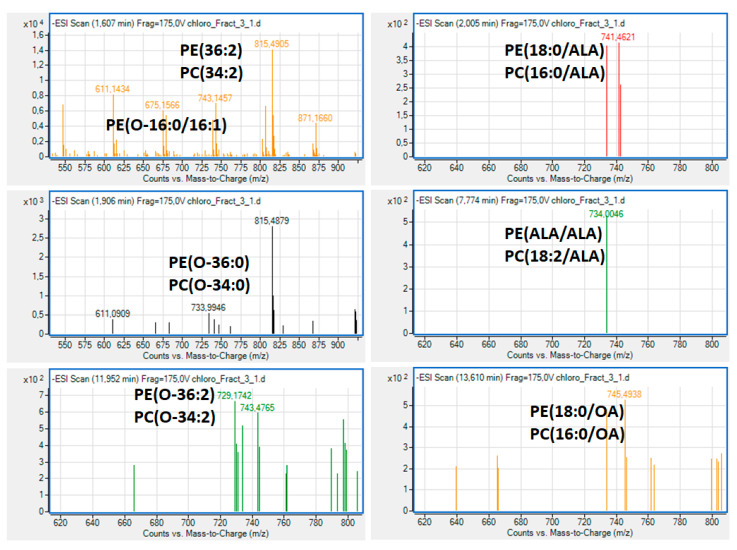
Representative mass spectra and proposed structures of the bioactive polar lipid molecules identified in the bioactive polar lipid fraction 3 of *Chlorococcum* sp., with strong anti-inflammatory and antithrombotic properties against the inflammatory and thrombotic mediators PAF and Thrombin in human platelets. PC = phosphatidylcholine; PE = phosphatidylethanolamine; ALA = α-linolenic acid (18:3 n-3); OA = oleic acid (18:1).

With respect to the LC-MS structural analysis of the bioactive polar lipid fraction 3 of *Clorococcum* sp. Phospholipids, survey scans in the negative ion mode between 600 and 1000 m/z of MS demonstrated that several phosphatidylcholine (PC) and phosphatidylethanolamine (PE) molecules were present, many of which were diacyl-PC and diacyl-PE molecules, More specifically, in this fraction several bioactive PC and PE molecules were identified (Figure 4), many of which were diacyl-PC and diacyl-PE molecules and less amounts of alkyl-acyl PC and alkyl-acyl-PE, respectively, bearing mostly SFA at the *sn*-1 position of their glycerol backbone and, at the *sn*-2 position, either the most abundant SFA (16:0 or 18:0) or MUFA (16:1 or the OA 18:1), and less but considerable amounts of such PL bearing that ALA n-3 PUFA.

Such bioactive PC and PE molecules bearing either the n-3 PUFA ALA or the MUFA OA, either found in several other natural sources, including marine sources [21,22,30], or specific standard molecules [36] with these structures, have been previously identified to possess strong antagonistic and agonistic effects against the PAF pathway of human platelet aggregation.

If present in foods and/or food supplements, after absorption, such dietary bioactive polar lipids (PC and PE molecules, with n-3 PUFA or MUFA at their *sn*-2 position) are usually delivered smoothly into plasma lipoproteins [14] and, from there, to several blood cells and tissues, including platelets, but also to tissues with accessibility issues, such as the brain. Their amphiphilic nature facilitates their journey within the blood stream and their incorporation into cell membranes and for surpassing the blood–brain barrier. After being transferred to blood cells, including platelets, these bioactive PC and PE molecules interact directly through a strong inhibitory antagonistic or a weak agonistic effect or both effects (in different concentrations) against the PAF and thrombin pathways of activating cells (including platelet aggregation) because of their structural resemblance to the PAF molecule and thus due to antagonism against the binding of PAF on its receptor [9].

Apart from their direct effect on the PAF-R, bioactive PL also beneficially modulates PAF metabolism in several cells, including platelets and leukocytes, and plasma, returning PAF levels and activities to homeostatic ones [9].

Some of these PC and PE molecules can also affect platelet aggregation indirectly due to their susceptibility to the enzyme activity of phospholipase A2 and the release of their bioactive MUFA and n-3 PUFA from their sn-2 position, which affect several thrombotic and inflammatory intracellular signalling pathways and gene expression [9]. For example, the released n-3 PUFA from such PE and PC molecules can beneficially affect the PAF/thrombin-induced inflammatory pathways of eicosanoids involved in platelet aggregation and other pro-inflammatory cascades by agonistically inhibiting the cyclooxygenases (COX), which are the basic enzymes involved in eicosanoid synthesis from arachidonic acid [9,12,13].

Therefore, the beneficial anti-inflammatory properties of such bioactive PC and PE molecules present in lipid fraction 3 of *Chlorococcum* sp. directly and/or indirectly protect against PAF and thrombin related inflammatory and thrombotic pathways. This further supports the putative health benefits of bioactive polar lipids of natural origin and especially from marine sources, such as those found in *Chlorococcum* sp. in the present study. However, further studies are needed to support such a notion, especially for using such microalga for the production of food supplements and nutraceuticals containing bioactive polar lipids with strong anti-inflammatory and antithrombotic properties against chronic disorders.

## 3. Materials and Methods

### 3.1. Microalgal Isolate

*Chlorococcum* sp. SABC 012504 (hereafter *Chlorococcum* sp.) was obtained from the Biobank at Shannon ABC, Limerick Institute of Technology. This marine microalga is one of the green microalgae belonging to the family Chlorococcaceae and was isolated from the coast of Ballybunion (Lat 52.511389, Lon 9.677496), Ireland. The microalga was maintained in artificial seawater nutrients III (ASN-III) medium (430 mM NaCl, 21 mM MgCl_2_, 7 mM KCl, 9 mM NaNO_3_, 0.11 mM K_2_HPO_4_, 29 mM MgSO_4_, 4.5 mM CaCl_2_, 15.6 µM Citric acid, 11 µM Ferric ammonium citrate, 1.5 µM EDTA (disodium salt), 189 µM Na_2_CO_3_, 46.25 µM H_3_BO_3_, 9.15 µM MnCl_2_, 0.77 µM ZnSO_4_, 1.61 µM Na_2_MoO_4_, 0.32 µM CuSO_4_ and 0.17 µM Co(NO_3_)_2_0 at pH 7.5–7.6 [37]. The microalga was incubated at low light intensity of 40 μmol photons m^−2^ s^−1^ with 16/8 h light/dark cycle at 20 °C.

### 3.2. Culturing of Microalga

**Figure 5:**
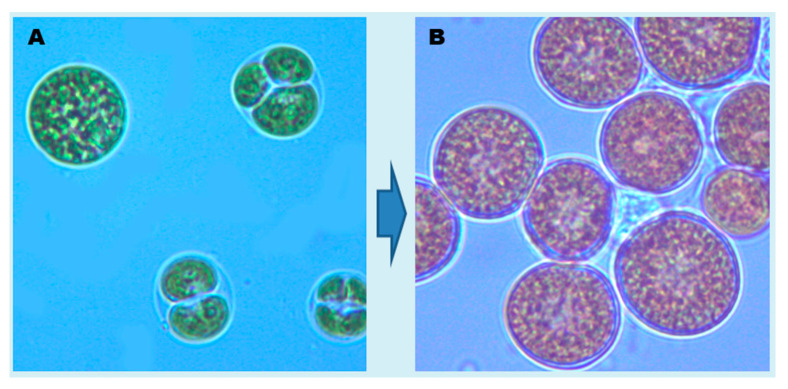
Photomicrographs showing the cells of *Chlorococcum* sp. from green phase (**A**) and stress phase (**B**) cultivation. During green phase the microalga grows actively as their dividing and mature cells are seen (**A**), while during stress phase, the microalga shows mostly static growth with cell enlargement and accumulation of lipids and carotenoids. The magnification of the photomicrographs was 1000 times (100 ȕ objective and 10 ȕ eyepiece).

The microalga was actively grown as green phase cultures in 250 mL Erlenmeyer flasks containing 100 mL of ASN-III medium for 10 days. Briefly, 500 μL of healthy green cells were inoculated per 100 mL growth medium to obtain an initial in vivo absorbance of cells of ~0.06 at 680 nm. The flasks were incubated in an environmental growth chamber at 20 °C, under the PAR (photosynthetically active radiation, 400–700 nm) illumination of 80 μmol photons m^−2^ s^−1^ for 16/8 h light/dark cycle. After, that green cells were harvested carefully and quickly by centrifugation at 3000× *g* at 20 °C for 4 min and were sub-cultured in ASN-III medium containing reduced amounts of NaNO_3_ (0.9 mM) and K_2_HPO_4_ (0.011 mM) for stress phase growth. The flasks were incubated at 20 °C, under the high-light PAR illumination of 200 μmol photons m^−2^ s^−1^ with 16/8 h light/dark cycle. Each day, flasks were hand shaken for synchronous stress phase cultivation (Figure 5). After two weeks of stress cultivation, biomass was harvested by centrifugation at 5000 rpm at 20 °C for 8 min and was stored at −20 °C until used for lipids extraction.

### 3.3. Bioactive Lipids Extraction and Fractionation

Extraction of lipids was carried out following the method of [38]. Briefly, a known amount of fresh biomass (1 g) was soaked overnight in 5 mL of extraction solvent (2:1 chloroform: methanol) at 4 °C. Then, the biomass was ground in a mortar and pestle by adding acid washed sand powder. The process of grinding was repeated by adding 10 mL of extraction solvent to ensure complete extraction of lipids. All extracts were pooled in a tube and made-up to 15 mL with extraction solvent, and 5 mL of ultrapure water was added. Then, the content was mixed gently by tube inversions to remove water-soluble impurities. Then the tubes was centrifuged at 5000 rpm for 6 min for the separation of two layers. The lower lipid layer was carefully transferred to a new tube where sodium sulphate crystals was added to eliminate the moisture. Then, the clear supernatants containing lipids were dried overnight in a fume hood and estimated the total lipid content gravimetrically.

The lipids were fractionated into neutral, glycolipids, and phospholipids as per the method described earlier [18]. Briefly, the dried lipids re-constituted in 2 mL of chloroform were loaded onto solid phase extraction (SPE) cartridge (Alumina neutral ALN 1 g/6 mL) packed with 2 mL bed volume of silica powder (pore size 60 Å, 230–400 mesh; Sigma Chemicals, Arklow, Ireland), and the flow-through was loaded once again to ensure maximum adsorption to the column. The flow-through obtained after 2nd loading to the column was considered as unbound flow through (FT) for testing bioactivity. Then, the column was eluted twice with chloroform: acetic acid (9:1, *v*/*v*) and considered as Fraction-1 (neutral lipids). Next, the column was eluted twice with acetone: methanol (9:1, *v*/*v*) and considered as Fraction-2 (glycolipids). Finally, the column was eluted twice with methanol and considered as Fraction-3 (phospholipids). Eluents at every fractionation stage were collected by centrifugation at 3000 rpm for 5 min. All the fractions were transferred in amber vials and dried with nitrogen flush.

### 3.4. Fatty Acid Composition and Structural Elucidation of Microalgal Lipid Fractions by LC-MS Analysis

The bioactive lipid fractions of *Chlorococcum* sp. against the PAF and thrombin pathways of human platelet aggregation, were analysed by LC-MS as previously described [21], in order to elucidate their overall structures and their saponified fatty acid composition (free fatty acids; FFA).

Briefly, each of these lipid fractions (flow through (FT), Fraction-1, Fraction-2, and Fraction-3 corresponding to the unbound, neutral lipid, glycolipid and phospholipids) was divided into two half parts and dried with N_2_ flush. The first half of each lipid fraction was saponified by adding 1.5 mL of saponification reagent (2.5 M KOH: methanol (1:4, *v*/*v*)) and by gentle vortex. Then the tubes were incubated at 72 °C for 15 min before the addition of 225 µL of formic acid. Then, 1725 µL of chloroform and 375 µL of ultrapure water were added to the tube, and vortexed to separate the content into two layers. The lower chloroform layer containing FFA was carefully transferred to amber gas chromatographyvials and evaporated to dryness and stored at −20 °C until used for LC-MS analysis.

For LC-MS analysis, all dried lipids were re-constituted in 500 µL of methanol: dichloromethane (2:1, *v*/*v*), centrifuged at 13,000 rpm for 6 min (Heraeus Biofuge Stratos, Fisher Scientific Ltd., Dublin, Ireland) and the content was filtered through 3 kDa ultra-centrifuge filters (Amicon Ultra 3k, Merck Millipore Ltd., Carrigtwohill, Co. Cork, Ireland). Then, 10 µL of the filtrate was injected and the fatty acid profiles were obtained from an HPLC (Agilent 1260 series, Agilent Technologies Ireland Ltd., Little Island, Co. Cork, Ireland) equipped with a Q-TOF mass spectrometer (Agilent 6520) and the source type was electrospray ionization (ESI). The column used for the resolution of fatty acids was an Agilent C18 Poroshell 120 column (2.7 µm, 3.0 × 150 mm). Mobile phase A consisted of 2 mM ammonium acetate in water and mobile phase B consisted of 2 mM ammonium acetate in 95% acetonitrile. Chromatographic separation was performed by gradient elution starting with 60% B for 1 min, then increasing to 90% B over 2.5 min. Subsequently, 90% B was held for 1.5 min and increased afterward to 100% over 5 min. Then, 100% B was held for 4 min, reducing afterward to 60% B over 0.5 min and held for 1 min until the next run. The mobile phase flow rate was 0.3 mL/min until 5 min elapsed, increasing up to 0.6 mL/min after 10 min and held at this flow rate until the end of the run. The mass spectrometer was operated in negative ionization mode, scanning from *m/z* 50–1100. Drying gas flow rate, nebuliser pressure and temperature were at 5 L min^−1^, 30 psi and 325 °C, respectively. Fragmentor and skimmer voltages were maintained respectively at 175 V and 65 V, and the capillary voltage was 3500 V. The monitoring reference masses used were 1033.988 and 112.9855 in the negative ion mode.

FFA and phospholipid species were assessed by a combination of survey, daughter, precursor, and neutral loss scans, while the identity of the bioactive lipids was verified using the LIPID MAPS: Nature Lipidomics Gateway (www.lipidmaps.org), based on the lowest delta values combined with the results obtained from the LC-MS analysis of the FFA that were produced by their saponification, as previously described [21].

### 3.5. Human Platelet-Rich Plasma (hPRP) Aggregation Studies of Microalgal Lipid Fractions

The evaluation of the anti-PAF and antithrombin effects of the bioactive lipids in human plasma rich in platelets (hPRP) was performed on a Chronolog-490 two-channel turbidimetric platelet aggregometer (Havertown, PA, USA), coupled to the accompanying AGGRO/LINK software package, as previously described [9,22,39]. Briefly, for hPRP isolation, healthy human volunteers (*n* = 10) donated fasting blood samples. The Ethics Committee of the University of Limerick approved the protocol and it was performed in accordance with the Declaration of Helsinki. Healthy donors were fully aware that their blood samples were used in our study and written consent was provided to the specialised phlebotomist. A total of 50 mL of blood was collected from the median cubital vein or cephalic vein of each healthy volunteer via venepuncture using a 20 G safety needle, and blood was drawn into sodium citrate anticoagulant S-Monovette using the aspiration method (0.106 mol/L in a 1:10 ratio of citrate to blood; Sarstedt Ltd., Wexford, Ireland). The collected blood samples were centrifuged at 194× *g* for 18 min at 24 °C with no brake applied, in an Eppendorf 5702R centrifuge (Eppendorf Ltd., Stevenage, UK). The supernatant hPRP was then transferred to polypropylene tubes at room temperature for the aggregation bioassays, whereas platelet-poor plasma (PPP) was obtained by further centrifuging the specimens at 1465× *g* for 20 min 24 °C with no brake applied. hPRP was adjusted to 500,000 platelets/mL if required by addition of the respective volume of PPP according to the absorbance of the hPRP measured in a Shimadzu UV-1800 spectrophotometer (Kyoto, Japan) using a quartz 1 cm cuvette. All procedures took place at 24 °C and all analyses were carried out within 2.5 h of the initial blood draw. PRP was stored at 24 °C before use in platelet aggregation bioassays.

Aliquots of standard PAF solution (Sigma Aldrich, Wicklow, Ireland) in chloroform/methanol (1:1 *v*/*v*) were evaporated under a stream of nitrogen and re-dissolved in bovine serum albumin (BSA) (2.5 mg BSA/mL saline; Sigma Aldrich, Wicklow, Ireland) to obtain PAF solutions with final concentrations into cuvette ranging from 2.6 × 10^−8^ to 2.6 × 10^−5^ mol/L. The examined PL samples were also dissolved in BSA (2.5 mg BSA/mL saline). Standard active thrombin (Sigma Aldrich, Wicklow, Ireland) was dissolved in saline prior testing. The ability of each selected sample to cause inhibition of either PAF-induced or thrombin-induced platelet aggregation was studied by adding various concentrations of each sample into the platelet suspension.

More specifically, prior to testing, 250 µL of hPRP was added to an aggregometer cuvette (Labmedics LLP, Abingdon on Thames, UK) at 37 °C with stirring at 1000 rpm and was calibrated prior to testing using the PPP as a blank. The maximum-reversible (or the minimum-irreversible) PAF-induced/thrombin-induced platelet aggregation was determined as the 100% aggregation, that was also used as baseline (0% inhibition), by adding PAF at approximately 2.6 × 10^−8^ M final concentration in the cuvette or active thrombin at approximately 0.01–0.04 U/mL in cuvette. The PAF-induced/thrombin-induced aggregation was calculated first at this 0% of inhibition baseline in a cuvette, whereas after the preincubation of hPRP with the test samples in a variety of concentrations, in a different cuvette the same amount of PAF/thrombin was added and the reduced aggregation was calculated, and thus a linear curve at the 20–80% range of the percentage of inhibition against PAF-induced/thrombin-induced aggregation of hPRP to the concentrations of each sample was deduced. From this curve, the concentration of the sample that led to 50% of PAF-induced/thrombin-induced aggregation of hPRP was calculated as the 50% inhibitory concentration value also known as the half maximal inhibitory concentration (IC_50_) value for each sample. All experiments were performed in triplicate (*n* = 3), using a different donors blood sample for each replicate, to ensure reproducibility. The resulting IC_50_ values were expressed as a mean value of the mass of lipid (µg) in the cuvette ± standard deviation (SD).

### 3.6. Statistical Analysis

Comparisons of bioactive lipids’ IC_50_ values against PAF-induced/thrombin-induced aggregation of human platelets were performed by the one-way analysis of variance (ANOVA) test. Differences were statistically significant when the *p* value was less than 0.05 (*p* < 0.05). The data were analysed using a statistical software package (IBM-SPSS statistics 26 for Windows, SPSS Inc., Chicago, IL, USA).

## 4. Conclusions

The in vitro assays of the present study showed that the glycolipid and phospholipid fractions of marine microalga *Chlorococcum* sp. SABC 012504 possess strong anti-PAF and antithrombin activities in human platelets. These bioactivities are likely due to the presence of specific novel *Chlorococcum* sp. SABC 012504 SQDG molecules, HexCer-t36:2 (t18:1/18:1 and 18:2/18:0) cerebrosides with a phytosphingosine base, phosphatidylcholine (PC), and phosphatidylethanolamine (PE) molecules in their lipid fractions. These bioactive PC and PE molecules with n-3 PUFA ALA or the MUFA OA were earlier reported from other natural sources, including marine, to possess strong antagonistic and agonistic effects against the PAF pathway of human platelet aggregation. Therefore, as evidenced in this study, the microalga *Chlorococcum* sp. has potential as a dietary supplement for these bioactivities.

## Figures and Tables

**Table 1:** List of microalgae, their growth conditions and lipid content.

Microalgal Species	Growth Conditions	Lipid Yield (% Dry Weight Biomass)	References
*Chlorella protothecoides*	Freshwater, heterotrophy-photoinduction	50.5	[23]
*Chlorococcum* sp. RAP13	Marine, heterotrophic	38.9 ± 1.9 *	[19]
*Chlorococcum* sp. RAP13	Marine, photoautotrophic	20.8 ± 2.6 *	[19]
*Chlorococcum* sp. SABC 012504	Marine, photoautotrophic	22 ± 2.52 *	This study
*Coelastrella* sp. F50	Marine, photoautotrophic	22 ± 1.7 *	[24]
*Dunaliella salina*	Marine, photoautotrophic, two-stage low-salt stress	43	[25]
*Dunaliella tertiolecta*	Marine, photoautotrophic, two-stage low-salt stress	40	[25]
*Haematococcus pluvialis*	Freshwater, photoautotrophic	32–37	[26]
*Haematococcus pluvialis* SCCAP K-0084	Freshwater, photoautotrophic	25–46 ± 1.25–2.3 **	[18]
*Isochrysis galbana*	Marine, photoautotrophic, two-stage low-salt stress	47	[25]
*Nannochloropsis oculata*	Marine, photoautotrophic, two-stage low-salt stress	29	[25]
*Porphyridium cruentum*	Marine, photoautotrophic	19.3	[27]
*Porphyridium purpureum*	Marine, photoautotrophic	9–14	[28]
*Tribonema* sp.	Freshwater, photoautotrophic, bacterial photoautotrophic co-cultivation	34.67–49.17	[29]

Note: *, ±SD; **, ±SE.

**Table 2:** Relative percentage of identified fatty acids of unsaponified (unsap) and saponified (sap) lipids of microalga *Chlorococcum* sp.

	Flow Through (FT) (R %)		Fraction-1 (R %)		Fraction-2 (R %)		Fraction-3 (R %)	
Fatty acids	Unsap	Sap	Unsap	Sap	Unsap	Sap	Unsap	Sap
Caprylic (C8:0)	1.75	0.00	0.56	0.00	1.06	0.00	0.00	0.00
Pelargonic (C9:0)	16.21	0.00	1.12	0.00	1.48	0.00	0.00	0.00
Lauric (C12:0)	0.00	0.00	0.00	0.00	0.00	0.00	0.00	0.05
Myristic (C14:0)	0.00	0.10	0.57	0.11	0.53	0.20	0.00	0.40
Pentadecylic (C15:0)	0.00	0.00	0.00	0.00	0.00	0.00	0.00	0.15
Palmitic (C16:0 (PA))	19.03	28.80	18.25	32.57	21.75	26.28	21.51	52.70
Palmitoleic (C16:1)	0.00	0.18	0.00	1.40	0.00	2.54	0.00	0.34
Margaric (C17:0)	0.00	0.00	0.00	0.00	0.00	0.38	0.00	0.58
Stearic (C18:0)	63.02	41.83	68.36	25.59	75.18	21.89	78.49	23.26
Oleic (C18:1 (OA))	0.00	22.74	9.91	21.55	0.00	20.86	0.00	13.47
Linoleic (C18:2 (LA))	0.00	1.64	0.00	11.76	0.00	18.30	0.00	2.72
Linolenic (C18:3 (ALA/GLA))	0.00	4.23	1.24	4.83	0.00	5.05	0.00	6.13
Stearidonic (C18:4)	0.00	0.15	0.00	0.74	0.00	1.11	0.00	0.19
Gadoleic (C20:1)	0.00	0.34	0.00	1.45	0.00	3.35	0.00	0.00
Dihomolinolenic (C20:3)	0.00	0.00	0.00	0.00	0.00	0.04	0.00	0.00
**Total number of SFAs**	**4**	**3**	**5**	**3**	**5**	**4**	**2**	**6**
**Total number of UFAs**	**0**	**6**	**2**	**6**	**0**	**7**	**0**	**5**
**Total peak area**	**1,421,451.59**	**149,087,839.98**	**6,085,702.65**	**284,509,430.70**	**3,285,702.02**	**265,139,703.41**	**1,211,926.85**	**171,329,929.52**

Note: R %, relative percentage of total peak area of identified fatty acids in a specific lipid fraction.

## Data Availability

The data presented in this study are available on request from the corresponding authors. The data are not publicly available due to being assessed with specific softwares for aggregometry and LC-MS that are not available to anyone, while a licence is needed to use them.
